# On Constructing Dynamic and Forward Secure Authenticated Group Key Agreement Scheme from Multikey Encapsulation Mechanism

**DOI:** 10.1155/2015/275496

**Published:** 2015-09-16

**Authors:** Iraj Fathirad, John Devlin

**Affiliations:** Department of Electronic Engineering, La Trobe University, Melbourne, VIC 3086, Australia

## Abstract

The approach of instantiating authenticated group key exchange (GAKE) protocol from the multikey encapsulation mechanism (*m*KEM) has an important advantage of achieving classical requirement of GAKE security in one communication round. In spite of the limitations of this approach, for example, lack of forward secrecy, it is very useful in group environments when maximum communication efficiency is desirable. To enrich this *m*KEM-based GAKE construction, we suggest an efficient solution to convert this static GAKE framework into a partially dynamic scheme. Furthermore, to address the associated lack of forward-secrecy, we propose two variants of this generic construction which can also provide a means of forward secrecy at the cost of extra communication round. In addition, concerning associated implementation cost of deploying this generic GAKE construction in elliptic curve cryptosystem, we compare the possible instantiations of this model from existing *m*KEM algorithms in terms of the number of elliptic curve scalar multiplications.

## 1. Introduction

A reliable and secure shared-key distribution scheme is arguably the most import step toward establishing any cryptographic channel among group of communicating parties. The group key exchange protocol (GKE) allows the members to calculate the shared-key over a public communication medium. An authenticated group key exchange (GAKE) scheme ensures that the resultant shared-key is kept indistinguishable to nonlegitimate peers and provides the participants with resistance against impersonation attack. A recently proposed approach [1] to achieve the classical requirement of authenticated key exchange security [2–4] for group scenario in one communication round is to construct it from the multikey encapsulation mechanism (*m*KEM). We refer to this generic framework by* mk*GAKE model. An* m*KEM [5] is a multipeer cryptographic solution that assumes receivers with long-term certified private/public-key pairs, and enables one entity to generate and efficiently encapsulate the same random session key for multiple receivers. The* mk*GAKE framework is basically constructed by parallel execution of a secure* m*KEM scheme among *n* parties. While this communicationally efficient GAKE construction provides the participants with basic requirements of key-confidentiality and impersonation resistance, it has two important limitations that can essentially affect the security and functionality of this model. These shortcomings and their undesirable effects are described below:

1. Lack of forward secrecy (FS): This implies compromising long-term keying materials of peers affects the confidentiality of previously established shared session keys. Since the existing* m*KEM solutions that used as building block of this framework are not FS, the resultant GAKE construction is not FS as well. FS is a desirable feature of a GAKE solution as it ensures the shared-key history remains confidential even after revealing the long-term keying materials of participants.
2. Inability to provide an efficient solution for dynamic group environments: This implies the participants need to reexecute the GAKE protocol when new members join or existing members leave. It is desirable for a GAKE protocol to provide an efficient mechanism for join/leave operations rather than reexecuting the scheme in dynamic group environment.

Another fundamental factor in analyzing the practicality of this framework is the computational cost of instantiating this generic model. Implementing this framework with the elliptic curve cryptosystem (ECC) can significantly reduce keys/parameters/messages size compared to the non-ECC variants [6]. It is therefore desirable to evaluate the ECC related deployment cost of this generic GAKE construction.

### 1.1. Our Contribution

In this paper we propose a generic framework to convert the existing static* mk*GAKE model to a partially dynamic scheme which provides a more efficient mechanism in the operation of joining new members to an already established session. In addition, to enrich the existing* mk*GAKE construction, we propose two variants of this model to achieve the important goal of forward secrecy at the cost of an extra communication round. As a further contribution, we evaluate the implementation cost of instantiating this model in ECC from existing* m*KEM schemes.

### 1.2. Related Works

Most of the group key exchange protocols in literature are based on either DH [11] or Joux [12] algorithms and require multiple rounds to establish the shared-key. Constructing a one-round, yet secure, GAKE is very desirable due to its appealing bandwidth efficiency compared to other multiround solutions. One of the first attempts to formalize one-round GAKE protocols was made in [13] that divides protocols into three different classes:

1. In the first class, peers are assumed to hold a preshared secret which is impractical and unreasonable in real environments [14].
2. In the second class one party encrypts its nonce to other participants together with digital signature on its encrypted nonce while other parties send their random values in the clear. An instantiation of such a scheme is given in [15]. It suffers from intensive computational overhead imposed by using digital signature as well as an inherent security concern resulting from unequal distribution of the key exchange responsibilities and giving extreme power to the encryptor party.
3. In the third class, which equally distributes the power and responsibility between participants, all peers encrypt their nonce to other entities using other participants' certified public-keys. A generic model to efficiently instantiate this class of one-round GAKE scheme from* m*KEM construction is given in [1] (referred to as* mk*GAKE model). So far, this* mk*GAKE model is the only practical one and provably secures one-round implicitly authenticated group key exchange construction in literature to date (while it is theoretically possible to construct a one-round GKE scheme by using multilinear map [16] and then converting it to an implicitly authenticated GAKE scheme in a similar way as MQV [17] or HMQV [18]; but, in spite of some recent improvements in constructing a plausible multilinear map [19, 20], these schemes are still far from being efficiently practical).

### 1.3. Organization

The next section discusses ECC and* m*KEM schemes and reviews the operation of dynamic GAKE protocols. In this section we also study the existing* m*KEM-based GAKE framework. In Section 3 we present our two variants of* m*KEM-based GAKE model with forward secrecy. In Section 4 we propose a generic framework to convert this existing static GAKE framework to a partially dynamic scheme. Finally, in Section 5 we compare the implementation cost of this framework from the possible ECC translation of existing* m*KEM solutions.  Section 6 gives a summary of our work and highlights the important points.

## 2. Preliminaries

### 2.1. Elliptic Curve

An elliptic curve *E* over the prime field of *F*_*q*_  (*characteristic*  (*F*_*q*_) ≠ 2, 3) is defined by a short Weierstrass equation *E*: *y* ^2^ = *x* ^3^ + *ax* + *b*, where the parameters *a*, *b* ∈ *F*_*q*_ are chosen such that Δ ≠ 4*a* ^3^ + 27*b* ^2^ (Δ is the discriminant of the equation). The group of points of *E* over *F*_*q*_ is denoted by *E*(*F*_*q*_) and the order of *E*(*F*_*q*_) is indicated by #*E*(*F*_*q*_). An elliptic curve is described by set of parameters (*q*, *a*, *b*, *P*, *p*, *h*), where *q* specifies the finite field of *F*_*q*_, *a* and *b* are coefficient of *E*, *P* = (*x*_*P*_, *y*_*P*_) is the generator of a cyclic subgroup of 〈*P*〉 ⊂ *E*(*F*_*q*_) of prime order *p*, and *h* = #*E*(*F*_*q*_)/*p* is the cofactor of elliptic curve. Elliptic curve DH assumptions are described as follows:

(i) A Diffie-Hellman (DH) tuple in *G* is (*P*, *xP*, *yP*, *zP*) ∈ *G* for some *x*, *y*, *z* ∈ *Z*_*q*_^*∗*^ satisfying *z* = *xy*mod⁡*q*.
(ii) *Computational Diffie-Hellman (CDH) problem*: given any three elements from the four elements in a DH tuple compute the remaining element.
(iii) *Decision Diffie-Hellman (DDH) problem*: given *P*, *xP*, *yP*, *zP* ∈ *G*, decide if it is a valid DH tuple or not (if *zP* = *xyP*).
(iv) *Hashed Decision Diffie-Hellman (HDDH) problem*: given *P*, *xP*, *yP* ∈ *G* and *h*, decide if *h* = *H*(*xyP*), where *H* is a target collision resistant Hash function.

### 2.2. *m*KEM

An* m*KEM scheme allows a peer to efficiently encapsulate a single session key to *n* parties. A typical *m*KEM scheme is presented by (*ℊ*_*m*_, *ℰ*_*m*_, *𝒟*_*m*_) tuples and consists of three core algorithms: private/public-key generation (*ℊ*_*m*_), key encapsulation (*ℰ*_*m*_), and key decapsulation (*𝒟*_*m*_). The probabilistic algorithm of *ℊ*_*m*_ takes domain parameters and generates public/private-key pairs (*pk*_*i*_, *sk*_*i*_). The probabilistic algorithm of *ℰ*_*m*_ takes set of public-keys *pk* = {*pk*_1_, …, *pk*_*n*_} of receivers and returns the encapsulation pair (*K*, *C*), where *C* = {*c*_1_, …, *c*_*n*_} and *c*_*i*_ is encapsulation of *K* with *pk*_*i*_. The deterministic algorithm of *𝒟*_*m*_ takes private-key *sk*_*i*_ and the encapsulation *C* and outputs *K*. For a *m*KEM scheme to be secure it is required to be sound, which means, for all key pairs (*pk*_*i*_, *sk*_*i*_) generated by *ℊ*_*m*_(*γ*) and all encapsulation pair (*K*, *C*) generated by *ℰ*_*m*_(*γ*, *pk*), we assume all possible range of *K* is generated by *𝒟*_*m*_(*γ*, *C*, *sk*_*i*_).

### 2.3. Dynamic Group Key Exchange

The GKE algorithms are divided into two groups of *static* and *dynamic* in terms of their capability to reform the session key with updated group membership. In static GKE the number of peers remains constant during the session whereas in dynamic GKE participants are allowed to join or leave the session at any time during the active session. A typical dynamic GKE consists of three algorithms, namely, shared-key establishment scheme,* join* operation, and* leave* operation. The shared-key establishment scheme operates the same as typical static GKE scheme and allows *n* parties to securely calculate confidential shared-key. The *join* operation allows new member to jointly establish new key with existing members in the way that the new member should not be able to extract the previously established session keys between those peers. The *leave* operation removes one of the members from the existing session and allows the remaining members to calculate a fresh key for the session. The leaving group members should be capable of calculating or distinguishing updated session key. Whilst it is possible to convert any static GKE to a dynamic GKE by reexecuting the static GKE with updated members, it is desirable for a GKE protocol to provide more efficient solution for join/leave operations rather than trivial approach of reexecuting the GKE scheme in dynamic environment.

### 2.4. *m*KEM-Based One-Round GAKE Construction (*m*kGAKE Model)

The generic model proposed by Gorantla et al. in [1] provides a framework to construct a one-round implicitly authenticated group key exchange (GAKE) from an* m*KEM scheme. Consider set of parties *𝒰* = {*u*_1_, …, *u*_*n*_} as participants in the GAKE scheme, where *u*_*i*∈[1, *n*]_ is the identity of a participant and *𝒰* is set of identities of all parties. This generic model assumes an IND-CCA secure *m*KEM(*ℊ*_*m*_, *ℰ*_*m*_, *𝒟*_*m*_) as the core algorithm and is designed to let the members of *𝒰* establish a shared session key through parallel execution *m*KEM(*ℊ*_*m*_, *ℰ*_*m*_, *𝒟*_*m*_). Since* m*KEM guarantees to the sender that only the legitimate receiver can decapsulate the session key, this generic model constructed from parallel execution of* m*KEM among multiple participants can provide all parties with implicit authentication on computed symmetric-key. This model consists of four phases as shown below:

(i) Initiation:
  (1) ui∈U:ski,pki⟵gmKEMD,pk=pk1,…,pkn  is  known  to  all  peers.
(ii) Computation:
  (2) ui∈U:Ki,Ci⟵EmKEMpkj ∣ pkj∈pk;  1≤j≠i≤n.
(iii) Communication:
  (3) $$\begin{array}{c} \left(\;u_{i}\in U\;\right)\text{ broadcasts }\left(\;u_{i},c_{i}\;\right)\text{ to }U\setminus \left\{\;u_{i}\;\right\}. \end{array}$$
(iv) Key calculation:
  (4) ui∈U  calculate  the  shared-key  as  followsKj1≤j≠i≤n⟵DmKEMski,Cj ∣ 1≤j≠i≤n,sid⟵C1||⋯||Cn||U,K⟵fK1sid⊕⋯⊕fKnsid.

In the* Initiation* phase, each GAKE participant *u*_*i*_ ∈ *𝒰* executes *ℊ*_*m*KEM_(*𝔻*) to obtain private/public-key pairs of (*sk*_*i*_, *pk*_*i*_), and authentic set of public-keys *pk* = {*pk*_1_, …, *pk*_*n*_} is known to all peers. In the* Computation* phase, each *u*_*i*_ ∈ *𝒰* executes* m*KEM encapsulation algorithm with other participants' public-key to obtain the symmetric-key and encapsulation pair. In the* Communication* phase each *u*_*i*_ ∈ *𝒰* broadcasts its computed encapsulation together with its id to all other peers. Finally, in the* Key calculation* phase each *u*_*i*_ ∈ *𝒰* executes the* m*KEM decapsulation algorithm on each of the incoming encapsulations using its private-key to obtain (*n* − 1) number of the symmetric-keys. Then, set sid to be the concatenation of all the incoming and outgoing exchanged messages sid ← (*C*_1_||⋯||*C*_*n*_||*𝒰*), where *𝒰* is the set of identities of all the users. Finally, sid and decapsulated keys are fed to a pseudorandom function to calculate the session key.

## 3. Two-Round GAKE with Forward Secrecy

The generic one-round* m*KEM-based framework cannot provide forward secrecy, but it can be extended to a two-round unauthenticated scheme to achieve this additional goal. In this approach, the authenticated and certified long-term private/public-key pairs are replaced with ephemeral key pairs. In the two-round variance, the participants execute the *m*KEM in parallel with on-demand generated ephemeral keys. Using ephemeral and uncertified asymmetric-keys will result in a GKE protocol without an implicit authentication property. In this case an adversary can impersonate any honest participant to other peers by replacing the ephemeral private/public values and resultant protocol is only secure in the presence of a passive adversary. To provide the GKE protocol with authentication, one of the two following approaches may be adopted.

### 3.1. Using Digital Signature in the First Round

In this approach the peer *u*_*i*_ ∈ *𝒰* is assumed to hold a certified long-term private/public-key pair of (*𝒮𝒦*_*i*_, *𝒫𝒦*_*i*_) corresponding to the employed digital signature scheme. The signing/verification algorithms of the corresponding digital signature scheme are denoted by (Sign, Verify). The key exchange procedure is carried out in two rounds as shown below.

*Round 1*. Peer *u*_*i*_ runs the *ℊ*_*m*KEM_ function to obtain ephemeral private/public-key pair of (*sk*_*i*_, *pk*_*i*_) and then use *𝒮𝒦*_*i*_ to compute the digital signature on *pk*_*i*_. Then, *u*_*i*_ broadcasts the signature together with its id and ephemeral public-key of *pk*_*i*_ to the other users. The generic framework for the first round interaction is shown below:

(i) Setup:
  (5) ui∈U:  obtains  long-term  signingverification  keys  of  SKi,PKi.The  verification  public-keys  PK=PK1,…,PKn  are  known  to  all  peers.
(ii) Initiation:
  (6) $$\begin{array}{c} \left(\;u_{i}\in U\;\right):\left(\;{sk}_{i},{pk}_{i}\;\right)⟵g_{mKEM}\left(\;D\;\right). \end{array}$$
(iii) Signature:
  (7) $$\begin{array}{c} \left(\;u_{i}\in U\;\right):\sigma_{i}⟵{Sign}_{{SK}_{i}}\left(\;u_{i},{pk}_{i}\;\right). \end{array}$$
(iv) Communication 1:
  (8) $$\begin{array}{c} \left(\;u_{i}\in U\;\right)\text{ broadcasts }\left(\;u_{i},\sigma_{i},{pk}_{i}\;\right)\text{ to }U\setminus \left\{\;u_{i}\;\right\}. \end{array}$$

*Round 2*. In the second round, other peers verify the authenticity of *pk*_*i*_ by validating the received signature using the publicly available certified verification key of *𝒫𝒦*_*i*_ and then run the one-round protocol with authentic ephemeral public-keys. The generic framework for the second-round interaction of this approach is shown below:

(i) Verification:
  (9) For  1≤j≠i≤n  peer  ui∈U  CheckVerifyPKjuj,pkj,σj=1.
(ii) Computation:
  (10) ui∈U:Ki,Ci⟵EmKEMpkj ∣ pkj∈pk;  1≤j≠i≤n.
(iii) Communication 2:
  (11) $$\begin{array}{c} \left(\;u_{i}\in U\;\right)\text{ broadcasts }\left(\;u_{i},c_{i}\;\right)\text{ to }U\setminus \left\{\;u_{i}\;\right\}. \end{array}$$
(iv) Key calculation:
  (12) ui∈U  calculate  the  shared-key  as  followsKj1≤j≠i≤n⟵DmKEMski,Cj ∣ 1≤j≠i≤n,sid⟵C1||⋯||Cn||U,K⟵fK1sid⊕⋯⊕fKnsid.

### 3.2. Using Digital Signature in the Second Round

A variant of this approach is (also) suggested in [21] and the core idea is originally borrowed from [22]. In this framework, peer *u*_*i*_ ∈ *𝒰* is assumed to have a pair of certified long-term signing/verification key pair of (*𝒮𝒦*_*i*_, *𝒫𝒦*_*i*_) corresponding to the employed digital signature scheme. The generic framework for the first-round interaction is shown below.

*Round 1*. In the first round, peer *u*_*i*_ runs the *ℊ*_*m*KEM_ function to obtain ephemeral private/public-key pair of (*sk*_*i*_, *pk*_*i*_) and broadcast it to other peers:

(i) Setup:
  (13) ui∈U:  obtains  long-term  signingverification  keys  of  SKi,PKi.The  verification  public-keys  PK=PK1,…,PKn  are  known  to  all  peers.
(ii) Initiation:
  (14) $$\begin{array}{c} \left(\;u_{i}\in U\;\right):\left(\;{sk}_{i},{pk}_{i}\;\right)⟵g_{mKEM}\left(\;D\;\right). \end{array}$$
(iii) Communication:
  (15) $$\begin{array}{c} \left(\;u_{i}\in U\;\right)\text{ broadcasts }\left(\;{pk}_{i}\;\right)\text{ to }U\setminus \left\{\;u_{i}\;\right\}. \end{array}$$

*Round 2*. In the second round, each *u*_*i*_ ∈ *𝒰* executes* m*KEM encapsulation algorithm with other participants' public-keys to obtain the symmetric-key and encapsulation pair. To provide the authentication property, *u*_*i*_ uses *𝒮𝒦*_*i*_ to compute digital signature on session key encapsulation *C*_*i*_ concatenated with ephemeral public-keys in the system and broadcast signature and *C*_*i*_ to other participants. Other peers verify the authenticity of received encapsulation of *C*_*i*_ (and corresponding embedded key) by validating the received signature using the publicly available certified verification key of *𝒫𝒦*_*i*_. After validating the authenticity of all received encapsulations, each peer extracts the embedded-keys by using* m*KEM decapsulation algorithm and finally computes the shared session key. The generic framework for the second round interaction is shown below:

(i) Computation:
  (16) ui∈U:Ki,Ci⟵EmKEMpkj ∣ pkj∈pk;  1≤j≠i≤n.
(ii) Signature:
  (17) $$\begin{array}{c} \left(\;u_{i}\in U\;\right):\sigma_{i}⟵{Sign}_{{SK}_{i}}\left(\;C_{i}\;|\;{pk}_{1}\;|\;\cdots \;|\;{pk}_{n}\;\right). \end{array}$$
(iii) Communication:
  (18) $$\begin{array}{c} \left(\;u_{i}\in U\;\right)\text{ broadcasts }\left(\;u_{i},\sigma_{i},C_{i}\;\right)\text{ to }U\setminus \left\{\;u_{i}\;\right\}. \end{array}$$
(iv) Verification:
  (19) For  1≤j≠i≤n  peer  ui∈U  CheckVerifyPKjuj,Cj ∣ pk1 ∣ ⋯ ∣ pkn,σj=1.
(v) Key calculation:
  (20) ui∈U  calculate  the  shared-key  as  followsKj1≤j≠i≤n⟵DmKEMski,Cj ∣ 1≤j≠i≤n,sid⟵C1||⋯||Cn||U,K⟵fK1sid⊕⋯⊕fKnsid.

### 3.3. Security Analysis

The provided security of both approaches relies on the security of the underlying digital signature scheme. Both approaches assume each peer *u*_*i*_ ∈ *𝒰* possesses a certified long-term private/public-key pair of (*𝒮𝒦*_*i*_, *𝒫𝒦*_*i*_) corresponding to the employed digital signature scheme. The relevant private-key, which is tasked to sign either ephemeral public-key (first approach) or encapsulation of the session key (second approach), should be kept secret as revealing this key to a potential adversary would result in revealing the session key. If we assume the employed signature scheme is secure against existential forgery under an adaptive chosen message attack, and the corresponding signing private-keys are kept secret from potential adversaries, then we can conclude both approaches are forward-secure authenticated group key exchange schemes. In fact, both approaches use a hierarchy of signatures where the first authenticated exchange authenticates the next exchange.

It should be noted that the ephemeral session keys are independent of the long-term keying materials, and the long-term keys are only tasked to authenticate the session key and not to take a role in the calculation of these keys. Thus, if an adversary manages to compromise a long-term keying material of any participating peers in a random session, he/she cannot reveal any information about the ephemeral keys of previous sessions in which the corrupted party has been participating in the past. However, the future session keys will not be secure against this adversary as he/she can fake the corrupted party and fool any other party(ies) to enter session key calculation phase with her/him. It should be noted that a forward-secure group key exchange is expected to keep the previous session keys unaccessible, not the future keys. Based on this notion, both of the described approaches are forward-secure key exchange schemes. Note that while the first approach (Section 3.1) is basically simpler and more convenient, the second method (Section 3.2) is stronger as it provides mean of mutual authentication on all the session-related ephemeral values.

## 4. Achieving Dynamic Group Operation

While it is possible to construct a dynamic protocol from this GAKE model by reexecuting (from scratch) the scheme in *join* or *leave* procedure, we propose a solution to perform the *join* operation more efficiently. The *leave* operation still requires the member to reexecute the protocol.

Consider a scenario where a new member *u*_*z*_, with knowledge of domain parameters *𝔻*, KDF, and *f*, decides to join an ongoing session between *𝒰* parties with the shared session key of *K*_*S*_. Peer *u*_*z*_ is required to run the *ℊ*_*m*KEM_(*𝔻*) function to obtain the private/public-key pair (*sk*_*z*_, *pk*_*z*_). The members of *𝒰* are required to have access to the certified public-key of *u*_*z*_. The members of *𝒰* also should have access to a secure symmetric encryption algorithm denoted by (*E*, *D*). To join the ongoing session, each of *𝒰* members together with *u*_*z*_ should follow the corresponding procedure as described below.

*Peer u*_*z*_. This new member executes the one-round protocol with respect to *𝒰* public-keys of *pk* = {*pk*_1_, …, *pk*_*n*_}:

(i) Initiation:
  (21) $$\begin{array}{c} \left(\;{sk}_{z},{pk}_{z}\;\right)⟵g_{mKEM}\left(\;D\;\right),\\ {pk}_{z}\text{ is known to all peers}. \end{array}$$
(ii) Computation:
  (22) $$\begin{array}{c} \left(\;u_{z}\;\right):\left(\;K_{z},C_{z}\;\right)⟵E_{mKEM}\left(\;\left\{\;{pk}_{1},\ldots ,{pk}_{n}\;\right\}\;\right). \end{array}$$
(iii) Communication:
  (23) $$\begin{array}{c} \left(\;u_{z}\;\right)\text{ broadcasts }\left(\;u_{z},C_{z}\;\right)\text{ to }U. \end{array}$$
(iv) Key calculation:
  (24) uz  calculate  the  shared-key  as  followsKi1≤i≤n⟵DmKEMskz,Ci ∣ 1≤i≤n,sid⟵C1||⋯||Cn||C1||⋯||Cn||Cz||U||uz,K⟵fK1sid⊕⋯⊕fKnsid⊕fKzsid.

*Peer u*_*i*_ ∈ *𝒰*. Each member of *𝒰* executes the one-round protocol with respect to *u*_*z*_ public-key of *pk*_*z*_ and use a symmetric encryption scheme with already established session key to distribute the new keys among themselves:

(i) Computation:
  (25) ui∈U:Ki,Ci⟵EmKEMpkz,Ci⟵EKsKi.
(ii) Communication:
  (26) ui∈U  broadcasts  ui,Ci,Ci  to  U∖ui∪uz.
(iii) Key calculation:
  (27) ui∈U  calculate  the  shared-key  as  followsKj1≤j≠i≤n⟵DKsCi ∣ 1≤j≠i≤n,Kz⟵DmKEMski,Cz,sid⟵C1||⋯||Cn||C1||⋯||Cn||Cz||U||uz,K⟵fK1sid⊕⋯⊕fKnsid⊕fKzsid.

It should be noted that in reexecuting the GAKE protocol from scratch each member needs to execute the associated* m*KEM protocol with public-keys of all existing and new members. However, with the help of the proposed framework, each of existing members of *𝒰* executes the associated* m*KEM protocol, in the joining of new member(s), only with public-key(s) of that (those) member(s) and does not include the public-keys of other existing members {*𝒰*∖*u*_*i*_}. Considering the expensive computational cost of* m*KEM schemes which is dependent on the number of inputted public-keys, this framework can significantly reduce the associated computational overhead in dynamic group environments. While this construction results in better efficiency compared to rerunning the algorithm in joining a new member, the security of this scheme solely depends on the security of the employed symmetric encryption scheme and security of the generic* m*KEM-based GAKE model (Section 2.4). The joining new peer of *u*_*z*_ executes the* m*KEM-based GAKE model with the existing members; thus, the security of the calculated key with this node is the same as the generic framework. Furthermore, other nodes of *u*_*i*_ ∈ *𝒰* execute the* m*KEM-based GAKE model with the new peer of *u*_*z*_ and, in the meantime, distribute their ephemeral values among themselves through a CCA2 symmetric encryption scheme; thus, the security of calculated session key with these peers relies on the security of the* m*KEM-based GAKE framework and employed symmetric encryption scheme, combined.

## 5. Efficiency Comparison of Instantiating GAKE Model from Different KEMs

In Table 1 we compare the efficiency of instantiating the* mk*GAKE model from existing provably secure* m*KEM schemes. The table compares the computation cost of such constructions in terms of number of associated EC point scalar multiplications which is denoted by SM. The SM calculation refers to computing *kP* where *k* is an integer and *P* is an EC point. The multiscalar multiplication denoted by MSM refers to computing ∑*k*_*i*_ *P*_*i*_. We found it easier and more consistent to represent the computational efficiency of different schemes by a single element of SM. However, since many factors contribute to computation of various MSM cases, then it is very difficult to precisely describe the computation cost of MSM in terms of calculation cost of SM. One approach to roughly estimate this relation, as described in [23], is by considering the unsigned binary representation of scalars and calculate MSM with a sliding window technique. An estimation from this approach is described in [24] and in windows size of 2 and bit-length of 256 it is assumed that one MSM calculation is roughly equal to 1.39 SM calculation. It should be noted that this optimistic estimation enables us to conveniently compare the computation efficiency of different EC-based schemes in a unified system.

## 6. Conclusion

Through this contribution, we propose an efficient and practical generic framework to convert static* m*KEM-based GAKE construction into a partially dynamic scheme. Our framework provides a more efficient solution for the join operation rather than the naive approach of reexecuting the original GAKE model with updated memberships. Furthermore, in order to enrich existing* m*KEM-based GAKE framework, we propose two variants of this generic model which can also provide a means of forward secrecy at the cost of an extra communication round. Finally, to evaluate the computational cost of deploying this generic model in elliptic curve cryptosystem, we compared the associated EC-related calculation cost of possible instantiations of this model from existing* m*KEM algorithms.

## Figures and Tables

**Table 1:** Efficiency comparison of GAKE construction from different *m*KEMs.

Scheme	Assumption	Initiation			Key exchange performance for *n* participants							
		SM	MSM	Key size *pk/sk*	Computation			Key calculation			Total ≈SM	Total KDF/Hash
					SM	MSM	KDF/Hash	SM	MSM	KDF/Hash		
PSEC [7]	CDH	1	—	2/1	*n*	—	*n* + 1/—	2*n* − 2	—	2*n* − 2/—	3*n* − 2	3*n* − 1/—
ELGamal [5]	CDH	1	—	2/1	*n*	—	1/1	2*n* − 2	—	*n* − 1/*n* − 1	3*n* − 2	*n*/*n*
CS98 [8]	DDH	1	2	5/5	*n* + 1	*n* − 1	1/*n* − 1	*n* − 1	*n* − 1	*n* − 1/*n* − 1	4.78*n* − 2.78	*n*/2*n* − 2
HK07 [9]	HDDH	3	—	4/3	2	*n* − 1	—/1	—	*n* − 1	—/*n* − 1	2.78*n* − 0.78	—/*n*
HTAS-CKS [10]	HDDH	—	3	5/5	2	2*n* − 2	—/2	2*n* − 2	2*n* − 2	—/2*n* − 2	7.56*n* − 5.56	—/2*n*
HTAS-HK [10]	HDDH	2	—	4/4	2	*n* − 1	—/2	—	*n* − 1	—/2*n* − 2	2.78*n* − 0.78	—/2*n*
